# Cytomegalovirus Disease in Renal Transplanted Patients: Prevalence, Determining Factors, and Influence on Graft and Patients Outcomes

**DOI:** 10.3390/pathogens10040473

**Published:** 2021-04-14

**Authors:** Carlo Maria Alfieri, Paolo Molinari, Mariateresa Gandolfo, Mariarosaria Campise, Donata Cresseri, Anna Regalia, Evaldo Favi, Min Li, Masami Ikehata, Serena Delbue, Piergiorgio Messa

**Affiliations:** 1 Department of Nephrology, Dialysis and Renal Transplantation, Fondazione IRCCS Ca’ Granda Ospedale Policlinico Milan, 20122 Milano, Italy; paolo.molinari1@unimi.it (P.M.); mariateresa.gandolfo@policlinico.mi.it (M.G.); maria.campise@policlinico.mi.it (M.C.); donata.cresseri@policlinico.mi.it (D.C.); anna.regalia@policlinico.mi.it (A.R.); piergiorgio.messa@unimi.it (P.M.); 2 Department of Clinical Sciences and Community Health, University of Milan, 20122 Milano, Italy; evaldo.favi@unimi.it; 3 Renal Transplantation, Fondazione IRCCS Ca’ Granda Ospedale Policlinico Milan, 20122 Milano, Italy; 4 Renal Research Laboratory Fondazione IRCCS Ca’ Granda Ospedale Policlinico Milan, 20122 Milano, Italy; li_min@libero.it (M.L.); masami.ikehata@policlinico.mi.it (M.I.); 5 Department of Biomedical, Surgical and Dental Sciences, Università degli Studi di Milano, 20122 Milan, Italy; serena.delbue@unimi.it

**Keywords:** cytomegalovirus, infection, renal transplantation, graft outcome, albumin

## Abstract

The prevalence and the factors related to cytomegalovirus (CMV) disease (CMVd) during the 1st year of renal transplantation (RTx) and the relationship between CMVd and early and long-term graft and RTx-patient (RTx-p) survival were evaluated. In 505 RTx-p, followed up for 8(5–11) years, data were recorded after 1-(T1) and 12-(T12) months of RTx. CMVd was defined either by CMV replication without clinical signs of disease (CMVr, 43%), or CMV replication with signs of disease (CMVs, 57%). During the 1st year of RTx, 45% of RTx-p had CMVd (CMVd+). CMVd+ patients were older than CMVd− patients. Female gender and Donor CMV-IgG+ (CMV IgG−D+)/recipient IgG- (CMV IgG−R-) status were more prevalent in CMVd+. At T1, CMVd+ had lower albumin, haemoglobin, and higher uric-acid and reactive C-protein than CMVd− and, at T1 and T12, received more steroids. Albumin-T1 was the unique factor in determining CMVd+, maintaining its significance also after the inclusion of IgG−D+/IgG−R− status to the model. CMVs had higher prevalence of CMV IgG-D+/IgG-R- than CMVr. CMVd, CMVr, and CMVs had no impact on graft loss (11% of RTx-p) and RTx-p death (8% of RTx-p). CMVd is highly prevalent during the 1st year of RTx. Albumin-T1 influences CMVd insurgence. CMVd did not impact on RTx and RTx-p loss.

## 1. Introduction

The prevalence of chronic kidney disease (CKD) has increased in recent years and renal transplantation (RTx) is considered the best therapy. Despite this, RTx patients (RTx-p) are characterized by a high risk of complications, partly related to their clinical characteristics and to therapies prescribed during the RTx. In this scenario, infectious diseases have a strong impact on graft and patient survival [1,2,3]

Cytomegalovirus (CMV) disease has a high prevalence in RTx-p, especially during the first year of RTx, ranging between 10% and 42% [4,5]. Generally, CMV disease (CMVd) may be classified according to the presence of clinical and biochemical signs in: asymptomatic CMV replication (CMVr) and symptomatic CMV replication (CMVs) [6]. Data present in literature have hypothesized a potential impact of CMVd on the negative outcome of RTx-p and RTx [7,8,9].

In our retrospective observational study, we evaluated the prevalence and the most important factors related to CMVd, the association between CMVd, and the principal long-term clinical outcomes in a cohort of RTx-p followed-up in our Department.

## 2. Results

### 2.1. Cohort Characteristics

The population examined in this study was composed of 505 RTx-p, of which Table 1 summarizes the main clinical characteristics.

The cohort was composed mainly of males (n = 292) and the median age of the cohort was 50 (41–58) years. Most RTx-p received hemodialysis (HD) before RTx (74%), while only 21% were treated with peritoneal dialysis (PD). Ninety-one percent of RTx-p received a kidney from a deceased donor. Among the 47 RTx-p who received a RTx from a living donor, 12 were transplanted from a related donor.

As shown in Table 2, in most cases, immunosuppressive induction therapy was composed of basiliximab and steroids (87%). The maintenance immunosuppressive therapy included calcineurin inhibitors, mainly Tacrolimus (88%), mycophenolate/mycophenolic acid (95%), and steroids (92%). At T1, only 3% of patients were treated with m-Tor inhibitors. This distribution showed no substantial differences at T12.

In Table 3, the main anthropometric and biochemical characteristics of the cohort studied at T1 and T12 are reported.

During the first year of RTx, a significant increase of body weight (*p* < 0.001) was found. No significant modification in blood pressure control was demonstrated.

Concerning renal function, serum creatinine (sCr) and daily uriniary protein excretion (Prot-U) were similar at T1 and T12. However, a significant reduction of estimated glomerular filtration rate (eGFR) (*p* < 0.0001) was found. In addition, a significant increase of uric acid, hemoglobin (Hb), serum albumin, and glycosylate hemoglobin (HbA1C) (all *p* < 0.0001) was observed.

#### CMV Evaluation

At the time of RTx, the most prevalent serological combination was CMV R+/D+ (74%), while CMV R−/D+ was present in 13% of RTx-p.

During the first year of RTx, CMVd was found in 225 RTx-p (45% of the cohort studied). Table 4 and Table 5 illustrate the main differences between patients who experienced CMVd (CMVd+) compared to those who did not (CMVd−) during the first year of RTx.

CMVd+ were older (*p* < 0.0001) and had a higher prevalence of diabetes before RTx (*p* = 0.004). In addition, in this group of RTx-p, the serological status CMV R−/D+ was significantly more represented (*p* = 0.01). Of note, CMVd- was significantly more prevalent in pre-emptive RTx-p (*p* = 0.01). No difference between the two groups was found in relation to dialysis vintage. RTx-p transplanted from a living donor were entirely represented in the CMVd− group.

Immunosuppressive therapy showed no associations with CMVd+ status, except for the cumulative dose of steroids, which was higher in the CMVd+ group, both at T1 (*p* = 0.006) and T12 (*p* < 0.0001).

At T1, the eGFR value was significantly lower in CMVd+ patients (*p* = 0.006). No difference was found in Prot-U. In addition, CMVd+ patients had significantly lower values of Hb (*p* = 0.02) and serum albumin (*p* < 0.0001), and higher values of uric acid (*p* = 0.01) and CRP (*p* = 0.008) at T1.

During the first year of RTx, the sCr showed no significant variation in either CMVd+ or CMVd− groups (CMVd+ T1: 1.39 (1.1–1.77)—T12: 1.39 (1.1–1.71) mg/dL *p* = 0.39; CMVd− T1: 1.37 (1.07–1.67)—T12: 1.30 (1.08–1.58) mg/dL *p* = 0.36). In the same period, in both groups, the eGFR significantly changed (CMVd+ T1: 49 (38–64)—T12: 49 (37–60) mL/min *p* < 0.0001; CMVd- T1: 55 (44–69—T12: 53 (43–65) mL/min *p* = 0.006).

In the multivariate analyses shown in Table 6 and Table 7, in which age at RTx, T1 serum albumin, Hb at T1, and cumulative dose of steroids at T1 were considered as dependent variables, only the serum albumin at T1 was independently and inversely correlated with the development of CMVd (*p* = 0.009—OR 0.50—IC 0.29–0.84). To confirm the strength of this relationship, it should be noted that the inclusion in the same model of CMV R-/D+ serologic status, which was also strongly and independently related with CMVd, did not significantly influence the association between serum albumin and CMVd (albumin T1: *p* = 0.008—OR 0.49—IC 0.28–0.82; serologic asset CMV R-D+: *p* = 0.01—OR 2.16—IC 1.18–3.95).

During the first year of RTx, among CMVd+ subjects, CMVr was reported in 97 patients (43%), while 128 patients (57%) have been considered to be suffering from CMVs.

Among CMVs, on medical indication, specific antiviral treatment was prescribed in 91% of cases, while, in 63% of cases, a significant CMV related reduction of immunosuppressive therapy was made. Of note, in 54% of cases, the two therapeutic approaches coexisted.

CMVr and CMVs groups were different only in the cumulative dose of steroids during the first year of RTx, significantly higher in CMVs (CMVr: 2722 (2595–3512) mg vs. CMVs 2902 (2674–3782) mg, *p* = 0.02).

### 2.2. Principal Outcomes

During the follow-up time, 58 patients (11%) re-started dialysis (D+), and 43 patients (8%) died. At T1 and T12, the sCr and Prot-U values were significantly higher in D+ (sCr T1: *p* < 0.0001—T12: *p* = 0.003; Prot-U T1: *p* = 0.004—T12: *p* < 0.0001). Consensually, at T1 and T12, eGFR was significantly lower in D+ (eGFR T1: *p* = 0.02—T12: *p* = 0.01). Finally, at T1 and T12, D+ had higher levels of uric acid (both *p* < 0.0001) and lower of Hb (Hb T1: *p* = 0.03—T12: *p* = 0.04). The serum albumin, on the other hand, showed lower values only at T12 in D+ (*p* = 0.02).

At the end of follow up, sCr > 50% was found in 18% of RTx-p and eGFRr > 50% in 15% of RTx-p; In the overall cohort, the median eGFR variation during the FU was −0.08 (−1.44/+1.11) mL/min/year.

### 2.3. Principal Long Term Outcome Related to CMVd

As shown in Table 8, no difference in graft failure and combined outcomes was found in relation to CMVd status.

This result was also confirmed by the analysis of eGFR variation during the FU in the two groups (CMVd+: −0.7 (−1.2/+1.1) mL/min/year vs. CMVd-: −0.9 (−1.6/+1.1) *p* = 0.66).

With the aim to evaluate the possible influence of a primary infection and of a reactivation in the principal outcome, a sub-analysis was performed. Globally, no statistical differences were found between those patients who experienced a first CMV infection and those who had CMV reactivation: D+ 7% vs. 9% *p* = 0.77; eGFRr > 50% + D+ 11% vs. 12% *p* = 0.96 and death D+ 7% vs. 9% *p* = 0.77. Additionally, considering the two subgroups alone, no impact of CMVd type was found. Briefly, in patients CMV R-, a first infection compared to no CMV infection did not influenced the main outcome considered: D+ 7% vs. 9% *p* = 056; eGFRr > 50% + D+ 11% vs. 13% *p* = 0.96 and death D+ 7% vs. 10% *p* = 0.57. In patients CMV R+, a CMV reactivation compared to no CMV reactivation did not influence the main outcome considered: D+ 9% vs. 13% *p* = 026; eGFRr > 50% + D+ 12% vs. 19% *p* = 0.06 and death D+ 7% vs. 9% *p* = 0.38.

**Figure 1:**
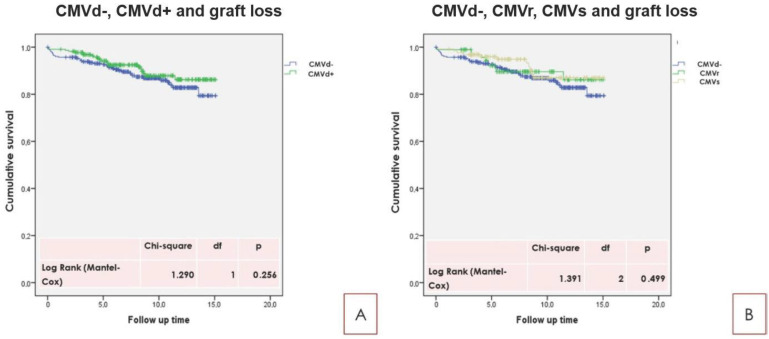
(**A**): CMVd and graft loss. (**B**): CMVd−, CMVr, and CMVs and graft loss. As reported in the text, no significant differences for CMVd, CMVe, and CMVs were found according to graft loss experience. Kaplan Meier survival analysis. The log rank test (Mantel-Cox) was used.

**Figure 2:**
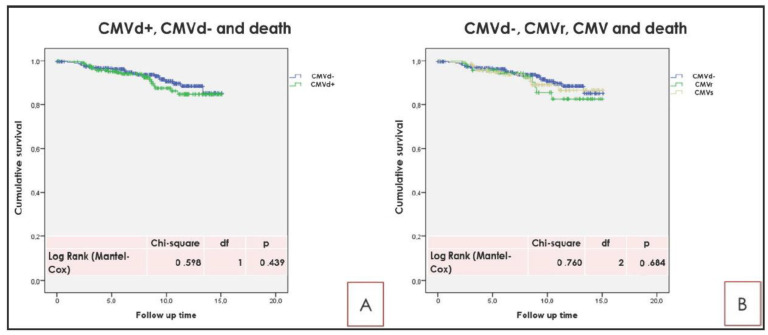
(**A**): CMVd and death. (**B**): CMVd−, CMVr, and CMVs and death. As reported in the text, no significant differences for CMVd, CMVr, and CMVs were found according to patient survival. Kaplan Meier survival analysis. The log rank test (Mantel-Cox) was used.

Survival analyses showed no influence of CMVd, CMVr, and CMVs on long-term RTx survival and on RTx-p survival (Figure 1 and Figure 2).

## 3. Discussion

The first aim of our study was to evaluate CMVd prevalence during the first year of RTx in a cohort of 505 RTx-p.

The prevalence of CMVd was 45%. Among CMVd+, 43% experienced a CMVr, whereas in 57%, CMVs was found. These results are in accordance with some data reported in the literature [10,11]. In the work presented by Witzcke et al., in which 296 RTx-p were studied, the prevalence of CMVd and CMVr was of 51% and 75%, respectively [12].

Concerning demographic characteristics, it is important to underscore that, in contrast with the principal international studies, in our cohort, CMVd had a significantly higher prevalence in the female gender. In some reports, a higher CMV female serum positivity in the Italian population has been described compared to the rest of the world. As reported in some Italian multicentric studies, CMV serum positivity in Italian women is >90% [13,14]. In Europe, this high prevalence was highlighted in Italy, Sweden, and, outside Europe, in Brazil [15].

The results of our study confirm the distribution of CMV serology among RTx-p, with a strong prevalence of the R+/D+ pattern (74%), followed by R−/D+ (13%), R+/D− (10%), and R−/D− (3%). In our cohort, the serological status mostly correlated with CMVd has been R−/D+. In agreement to our results, Kute et al., in a study of 750 RTx-p, indicated the R−/D+ status as one of the principal risk factors for the development of CMVd [16]. In addition, Selvey et al. confirmed the independent association between CMV R−/D+ and CMVd with an hazard ratio of 5.44 (2.49–11.89) [17].

Our study also aimed to assess the most impacting factors on CMVd development during the first year of RTx. From the comparison between CMVd+ and CMVd-, age was directly correlated to CMVd+. This could result from different components, partly mutually related. Certainly, a global cellular senescence might influence the general responsiveness of the immune system, as reported in the study of Arthurs et al. [18]. In agreement with this, in another study where 1127 RTx-p were considered, a Charlson comorbidity index >3 was strictly correlated to the development (over 3 months after the RTx) of CMVs [18]. Another important factor might be represented by the fact that the allocation to older patients of potentially marginal organs, theoretically more immunogenic, requires stronger immunosuppressive protocols.

In our cohort, CMVd was found more frequently in patients who underwent dialysis independently of dialysis modality and vintage. Several studies have reported a relationship between uremic toxins and oxidative stress. This, in addition, negatively influences the immune responsiveness, and might affect the patient’s nutritional and health status [19,20]. As mentioned above, our data showed no correlation between dialysis vintage and CMVd development. The impact of dialysis vintage on CMVd is still debated. In 2002, Abbot et al. evaluated in 33749 RTx-p the factors mostly related to hospitalization for CMV. According to their results, history of dialysis and dialysis vintage were independently associated with the outcome considered [21]. More recently, however, Corivaud et al. found no difference in CMVs frequency in 2010 RTx-p regarding dialysis technique and vintage [22].

Another important data from our analyses is the total absence of CMVd in RTx-p transplanted from living donors. A low CMVd+ prevalence was described in the literature in some papers. In particular, in a cohort of 592 RTx-p (214 RTx-p % and from living donors), Giakoustidis et al. reported a prevalence of CMVd+ of 7% and 12% respectively in living and deceased donor RTx [23]. The procedure frequently carried out before the start of replacement therapy, the better donor and transplant status, and the short ischemia time of the transplanted organ might be important factors in explaining this result [24]. Another possible explanation might be derived from the fact that related recipient and donor might have been infected with the same type of CMV, having antibodies against the same CMV serotype. However, in our cohort, most of RTx living donors were non related to recipients.

Interestingly, and in accordance with some reports, CMVd+ patients had higher prevalence of pre-RTx diabetes. In this regard, basic research studies indicate a potential promoting role, through a reduction in cell-mediated immunity, for diabetic status in CMVd development [25].

Concerning immunosuppressive therapy, the cumulative dose of steroids was found to be the only related factor in the onset both of CMVr and CMVs. This might be due to the effects of steroids on immunity (influence on activation, proliferation and apoptosis of T-cells; modulating effects on the NF-kb system) and in CMV reactivation mechanism (mediated by the same NF-kb pathway) [26,27,28,29].

Finally, in our study, Anti-thymocyte globulin (ATG) induction therapy was not associated with a higher frequency of CMVd. This topic is debated, considering CMV prophylaxis protocols frequently used in ATG treated RTx-p [30,31]. However, the strong lymphocytic depletion derived by the use of these antibodies might theoretically increase the risk of CMV replication [32]. It is important to point out that the prescription of ATG based induction protocols has increased strongly in the last 4–5 years in our Department and this might be a bias on this analysis. Concerning therapy with inhibiting mTORs, a statistically significant difference between CMVd+ and CMVd- was found at T1. In 2011, Brennan et al. reported, in a study of 2004 patients, a significantly reduced incidence of CMVd+ in those who had used high dosages of m-Tor inhibitors [32].

According to our data, one of the strongest and inversely related factors to the development of CMVd is serum albumin. In patients with CKD and RTx, albumin is considered an indirect indicator of inflammatory, as well as nutritional status [33,34,35]. Basic research data showed that albumin has a scavenger function of various endotoxins and oxidizing factors such as nitric oxide and its relationship with CMVd might be explained by the link between global inflammation and the activation of the NF-kb pathway, which can promote CMV replication [36,37,38]. Moreover, Liu et al. recently reported that CMV is able to downregulate the neonatal Fc receptor, involved in albumin level maintenance via US11. This might influence the relationship between CMV replication and serum albumin [39].

In addition, some evidence has reported the hypothesis that the pharmacological activity of mycophenolate mofetil (MMF), partially bounded to albumin, is a function of unbound drug concentration. This might imply a higher unbound MMF concentration in those patients with lower albumin levels, and consequently a higher degree of immunosuppression exposition [40,41].

The correlation between serum albumin levels and CMVd in RTx has been recently investigated. Srivastava et al., in their work published in 2020, enrolled 1717 RTx-p with the aim of studying the correlation of pre-transplant albumin levels and CMVd during RTx. In this study, severe pre-RTx hypoalbuminemia was associated with an increase incidence of post RTx CMVd and the dose–response relationship showed a slight decrease in CMVd risk with higher albumin levels [42]. In addition to that, some evidence is reported in liver transplantation. In 2010, Kim et al. studied 353 liver transplant patients for an average follow-up of 13 years. Data obtained in their study showed that patients who were found to have CMV replication had significantly lower values of albumin and Hb. Of note, also in that work, serum albumin was independently and inversely associated to CMVd [43].

Considering the several discordant results present in the literature concerning CMVd and early and long-term RTx and RTx-p outcomes, we decided to also explore this topic. During the FU time, 11% of RTx-p restarted dialysis, and 8% died. The incidence of the outcomes considered was significantly lower than the general European and American data. In fact, in Europe, the average survival rate of 5-year transplantation is 80%, with mortality rates always at 5 years of 9%, and in the USA, the 10-year graft survival rate is 35% and the RTx-p mortality rate, 36% [44,45].

From the analyses presented in our work, CMVd, CMVr, and CMVs showed no significant association with the major clinical outcomes evaluated. These results are partially in contrast with some data reported in the literature. In particular, Sagedal et al., in 2004, analyzing a cohort of 471 RTx-p for 7 years, found a correlation between CMVd and mortality for all causes, and an impact only of CMVs on graft failure. Differently to the clinical practice of our Department, in that study, none of the patients considered received CMV prophylaxis and, after the first 3 months of RTx, patients were reevaluated for CMV-DNA once a year [46]. More recently, another study performed by Aziz et al., in 2020, in 757 kidney–pancreas transplant patients, found a negative correlation between CMV infection and all cause kidney graft survival. It should be noted, however, that in this study, CMV viremia was not checked during CMV prophylaxis unless there was clinical suspicion of CMV disease; moreover, CMVd was not well defined and diagnosis of viral activation was made using a “diagnosis code”. Therefore, there was no data on the type and the severity of CMVd in that cohort. On the other hand, two important studies, both by Erdbrugger et al., showed results in a cohort of 594 kidney–pancreas transplant patients that were closely similar with many of our findings. Of note, CMV prophylaxis protocols adopted in those cohorts were almost identical to the one used in our Center. In these studies, the multivariate analyses indicated a strong influence both of higher donor age and of worse early post-RTx graft function in determining graft loss. CMV viremia or disease were not significantly related with graft outcome, detection of chronic rejection, and patient survival [47,48]. In addition, in our study, other minor and combined clinical outcomes were also considered in statistical assessments (eGFRr > 50% to FU, sCr > 50% to FU, outcome combined sCr > 50%—D+ and eGFRr > 50%—D+, and overall reduction of eGFR weighted for the time of FU), but no correlations with CMV were found.

Our study presents, as its principal limitations, the monocentric and the retrospective observational design and this has not allowed the evaluation of any causal relationships derived from the demonstrated associations and a more detailed investigation concerning the relationship between CMVd and inflammatory status (IL-6, MCP-1, etc.). Moreover, the fact that different dosage methods were adopted for CMV-DNA viral load from 2004 to 2017 in our Laboratory did not allow a focused statistical analysis. However, the high number of patients considered, and the relatively long FU time are, in our opinion, important features. In any case, this work might promote future randomized and prospective studies on the topics proposed.

In conclusion, our study demonstrated a relatively high prevalence of CMVd during the first year of RTx. Serum albumin at T1 was the most important independent factor which could influence CMVd insurgence. Nevertheless, CMVd did not show any relationship with principal RTx and RTx-p outcomes.

## 4. Materials and Methods

### 4.1. Study Design and Characteristics

In our study, data concerning 505 RTx-p were collected. The cohort studied was selected, among the 616 RTx-p transplanted in our Department between January 2004 and December 2016, according to the availability both of donor (D) and recipient (R) CMV serology at the moment of RTx and the achieving of one year RTx follow-up (6 and 2 patients restarted dialysis and died before the 12th month of RTx). The studied cohort, followed up for a minimum time of 12 months and a global median time of 8.3 years, was similar in the main clinical and biochemical characteristics to the one excluded from the study.

At RTx, for each of the 505 RTx-p examined, the following data were considered:—General characteristics: age, gender, dialysis vintage and technique, native renal disease, history of diabetes mellitus before RTx, steroid use before RTx, hepatitis C virus serology, cold ischemia time, type of RTx (living/deceased donor);—At 1st (T1) and 12th (T12) month after RTx: body weight, systolic and diastolic blood pressure; routine laboratory assessment including serum creatinine (sCr), estimation of glomerular filtration rate (eGFR), hemoglobin (Hb), serum albumin, blood glucose, glycated hemoglobin (HbA1C), uric acid, C-reactive protein (CRP), 24-h protein urinary excretion (Prot-U);—In addition, data about: immunosuppressive therapy regimens were evaluated at T1 and T12; steroid therapy was considered as total drug exposure, in mg, at T1 and T12.

Urinary and blood evaluations were performed after 12 h of fasting at the Transplant Clinic of Our Department and analyzed by the Central Laboratory of our Hospital.

All other biochemical parameters were measured according to the routine methodology used in our central laboratory. The sCr assessed was done by Jaffe’s reaction, whereas eGFR was estimated using the Modification of Diet in Renal Disease (MDRD) formula [49]. Urinary protein excretion was assessed by measuring the 24-h urinary collection protein through the immunoturbidimetric method.

Given the observational and retrospective nature of the study, it was not necessary to obtain informed consent from patients. In any case, all the data were collected digitally, analyzed, and reported in the results in a totally anonymous manner.

### 4.2. Cytomegalovirus Analysis 

According to CMV IgG serology, assessed at the moment of RTx both in R and D, four groups for CMV serology were defined: recipient CMV IgG−/donor CMV IgG− (CMV R−/D−); recipient CMV IgG-/donor CMV IgG+ (CMV R-/D+); recipient CMV IgG+/donor CMV IgG− (CMV R+/D-); recipient CMV IgG+/donor CMV IgG+ (CMV R+/D+).

In agreement with the clinical practice of our Department, prophylactic therapy for CMV, using valganciclovir at dosage corrected for renal function, was administered during the first three months after RTx to those patients considered at high risk of CMVd (CMV R-/D+, patients treated with ATG induction therapy, previous history of RTx).

During the first year of RTx in all the cohort, CMV viremia (CMV-DNA) was tested in all the RTx-p with the following frequency: within the first month of RTx: one to three times a week; from two to four months of RTx: every 10 days; from 4 to 6 months of RTx: every 15–20 days; from six months to 12 months after RTx: monthly.

According to the different dosage methods adopted for CMV-DNA from 2004 to 2017, the following cut-off values of CMV-DNA were used to define the presence of CMVd: from 2004 to 2006: CMV-DNA > 40 copies; from 2007 to the end of 2016: CMV-DNA > 500 copies; from 2017: CMV-DNA > 135 copies.

Patients with positive CMV-DNA, in the absence of clinical and biochemical signs of disease and in which no modifications of immunosuppressive and antiviral therapy were necessary, were defined as replicant CMVd (CMVr). Patients with positive CMV-DNA, associated to CMV clinical and/or biochemical signs of disease (for instance CMV related leucopenia and/or increase of liver necrosis indices) and in which a reduction of immunosuppressive therapy (for instance mycophenolate suspension) and/or a treatment with specific antiviral drugs were prescribed, have been defined as symptomatic CMVd (CMVs).

### 4.3. Outcomes and Follow up

Patients were followed up for a median time of 8.3 (5.4–11.4) years.

At the end of follow up (FU), the following outcomes were evaluated: increase of sCr > 50% compared to T1(sCr > 50%); reduction of eGFR > 50% compared to T1 (eGFR > 50%); reduction of eGFR/year: (mL/min)/year; Graft loss, defined by the need of restart of dialysis; RTx-p death. The following combined outcomes were also considered: graft loss or increase in sCr > 50% compared to T1; graft loss or eGFR > 50% reduction compared to T1.

### 4.4. Statistical Analyses

Continuous variables were expressed as median (25–75% ile). Differences between the groups were determined using the Student *t*-Test, the Wilcoxon–Mann–Whitney test, Kruskal Walls, ANOVA, the Chi square test, and the Fisher test when indicated. Logistic regression models were performed for multivariate analyses. The Kaplan–Meier test with log rank significance tests was used for survival analyses. Statistical significance was set for *p* < 0.05 values. Statistical analyses were performed using Statistica version 10 and SPSS 20.

## Figures and Tables

**Table 1:** Main general characteristics of the cohort studied.

Parameters	
**Number of patients** **N**	505
**Age at RTx** (years)	50 (41–58)
**Gender** (M-F) **N**	292–213
**Native kidney disease**	
**N** (%)	
**GNF**	106 (21)
**GNC**	94 (18)
**ADPKD**	92 (18)
**OTHER**	89 (18)
**CNP**	49 (10)
**NDD**	46 (9)
**VASCULITES**	26 (5)
**UES**	3 (1)
**Dialysis type** (HD-PD-No)	
**N** **(%)**	372-105-28 (74-21-5)
**Dialysis vintage** (months)	52 (37–76)
**RTx type**	
(deceased-living donor)	
**N** **(%)**	458-47 (91-9)
**Number of RTx**	
(1-2-3)	
**N** **(%)**	412-89-4 (81-18-1)
**Cold ischemia time** (hours)	13 (11–16)
**Diabetes at RTx**	
**N** **(%)**	23 (4)
**Steroid therapy before RTx**	
**N** **(%)**	189 (38)
**CMV Receiving-Donor Serology**	
**N (%)**	
**CMV R−/D−**	17 (3)
**CMV R−/D+**	64 (13)
**CMV R+/D−**	52 (10)
**CMV R+/D+**	372 (74)
**HCV+ before RTx**	
**N** **(%)**	31 (6)

Note: N: Number; RTx: renal transplantation; M: male; F: female; GNF: glomerulonephritis; GNC: chronic glomerulonephritis; ADPKD; autosomal dominant polycystic kidney disease; CNP: chronic pyelonephritis; NDD: non determined disease; UES: uremic emolytic syndrome; HD: hemodialysis; PD: peritoneal dialysis; CMV: cytomegalovirus; R: recipient; D: donor; HCV: hepatitis C virus.

**Table 2:** Immunosuppression therapy of the cohort studied.

Drugs	T1	T12
**Immunosuppressive induction therapy**		
**Basiliximab N (%)**	442 (87)	NA
**ATG N (%)**	58 (11)	
**No Basiliximab—No ATG N (%)**	5 (2)	
**Maintenance immunosuppressive therapy**		
**MMF-MPA-Tac-Steroids-CyA-m-Tor-I**	443-479-463-60-15	440-407-458-56-34
**N (%)**	(95-88-92-12-3)	(80-87-91-12-7)
**Cumulative dose of steroids (mg)**	880 (840–1050)	2722 (2598–3223)

Note: N: number; ATG: antithymoglobuline; CyA: Ciclosporine; MMF-MPA: mycophenolate-mycophenolic acid; Tac: tacrolimus; m-Tor-I: m-Tor inhibitors.

**Table 3:** Anthropometric and biochemical characteristics of the cohort studied.

Parameters	T1	T12	*p*
**Body Weight (Kg)**	65 (56–73)	68 (57–75)	**<0.001**
**SBP (mmHg)**	130 (120–140)	130 (120–140)	0.7
**DBP (mmHg)**	80 (75–90)	80 (75–85)	0.59
**s-Creatinine (mg/dL)**	1.38 (1.09–1.7)	1.33 (1.1–1.61)	0.21
**Prot-U (g/24h)**	0.203 (0.143–0.300)	0.175 (0.113–0.251)	0.43
**eGFR (mL/min)**	53 (40–67)	51 (41–63)	**<0.0001**
**Uric acid (mg/dL)**	5.8 (4.8–6.9)	6.4 (5.5–7.5)	**<0.0001**
**Hb (g/dL)**	11 (10.05–12)	12.7 (11.8–13.8)	**<0.0001**
**s-Albumin (g/dL)**	4.2 (4.4–4.5)	4.5 (4.2–4.7)	**<0.0001**
**Blood glucose (mg/dL)**	82 (72–93)	82 (74–94)	0.54
**HbA1C (%)**	5.5 (5.1–5.9)	5.7 (5.4–6.1)	**<0.0001**
**CRP (mg/dL)**	0.25 (0.1–0.64)	0.13 (0.1–0.390)	**0.001**

Note: SBP: systolic blood pressure; DBP: diastolic blood pressure; s-: serum; Prot-U: Proteinuria; eGFR: estimated glomerular filtration rate; Hb: hemoglobin; HbA1C: glycosylated hemoglobin; CRP: C-reactive protein.

**Table 4:** General characteristics of the cohort studied according to CMVd status.

Parameters	CMVd+ (N = 225)	CMVd- (N = 280)	*p*
**Age** (years)	53 (45–59)	47 (39–57)	**<0.0001**
**Dialysis vintage** (months)	48 (26–75)	51 (36–71)	0.103
**Cold ischemia** (hours)	13 (11–16)	14 (11–16)	0.266
**Gender M-F**	115–110	177–103	**0.0062**
**Native kidney disease, N**			0.882
**GNF**	46	59	
**ADPKD**	42	49	
**OTHER**	43	47	
**GNC**	40	53	
**CNP**	25	24	
**NDD**	19	29	
**VASCULITIS**	9	17	
**UES**	1	2	
**Dialysis type** (HD-PD-No)	171-49-5	201-56-23	**0.01**
**RTx type** (deceased-living)	225-0	233-47	**<0.0001**
**Number of RTx** (1-2-3)	193-32-0	218-58-4	**0.023**
**BAS-ATG Induction Therapy**	194-26	248-32	0.889
**Calcineurin Inhibitors T1** (Tac-Cya-No)	199-25-1	244-36-0	0.412
**T1 MMF** (Yes-No)	218-7	261-19	0.070
**T1 m-Tor-I** (Yes-No)	3-222	14-266	**0.015**
**Calcineurin Inhibitors T12** (Tac-Cya-No)	189-29-7	233-34-13	0.746
**T12 MMF** (Yes-No)	209-16	250-30	0.132
**T12 m-Tor-I** (Yes-No)	13-212	25-255	0.284
**Diabetes at RTx** (Yes-No)			**0.0046**
**N** **(%)**	18-207 (8–92)	6-274 (2–98)	
**Steroid therapy before RTx** (Yes-No)			0.343
**N** **(%)**	88-137 (39–61)	118-162 (42–58)	
**HCV+ before RTx** (Yes-No)			0.576
**N** **(%)**	14-211 (6–94)	22-258 (8–92)	
**CMV Recipient/Donor Serology**			**0.01**
**N**			
**CMV R−/D−**	4	13	
**CMV R−/D+**	38	26	
**CMV R+/D−**	18	34	
**CMV R+/D+**	165	207	

Note: N: number; M: male; F: female; GNF: glomerulonephritis; ADPKD: autosomal dominant polycystic kidney disease; GNC: chronic glomerulonephritis; CNP: chronic pyelonephritis; NDD: non determined disease; UES: uremic emolytic syndrome; HD: hemodialysis; PD: peritoneal dialysis; RTx: renal transplantation; BAS: Basiliximab; ATG: antithymoglobuline; Tac: Tacrolimus; CyA: Ciclosporine; MMF-MPA: mycophenolate-mycophenolic acid; m-Tor-I: m-Tor inhibitors; HCV: hepatitis C virus; CMV: cytomegalovirus; R: recipient; D: donor.

**Table 5:** Anthropometric and biochemical characteristics of the cohort studied according to CMVd status.

Parameters T1	CMVd+ (N 225)	CMVd- (N 280)	*p*
**Body weight (Kg)**	65 (55–73)	64.5 (56.7–73.05)	0.846
**SBP (mmHg)**	130 (120–145)	130 (120–140)	0.819
**DBP (mmHg)**	80 (70–90)	80 (75–90)	0.471
**s-Creatinine (mg/dL)**	1.39 (1.1–1.77)	1.37 (1.07–1.67)	0.225
**Prot-U (g/24h)**	0.198 (0.140–0.304)	0.210 (0.151–0.299)	0.624
**eGFR (mL/min)**	49 (38–64)	55 (44–69)	**0.006**
**Uric acid (mg/dL)**	6.1 (5.0–7.2)	5.7 (4.7–6.7)	**0.015**
**Hb (g/dL)**	10.80 (9.9–11.8)	11.1 (10.3–12.1)	**0.026**
**s-Albumin (g/dL)**	4.2 (3.9–4.3)	4.3 (4.03–4.50)	**<0.0001**
**Glycemia (mg/dL)**	84 (72–94)	81 (71.25–93)	0.123
**HbA1C (%)**	5.5 (5.1–5.97)	5.45 (5–5.8)	0.125
**CRP (mg/dL)**	0.30 (0.10–0.85)	0.2 (0.09–0.55)	**0.008**
**Cumulative steroids during the first year of transplantation (mg)**	890.00 (840.00–1125.00)	870 (835–995)	**0.006**

Note: SBP: systolic blood pressure; DBP: diastolic blood pressure; s-: serum-; Prot-U: proteinuria; eGFR: estimated glomerular filtration rate; Hb: hemoglobin; HbA1C: glycosylated hemoglobin; CRP: C-reactive protein.

**Table 6:** Multivariate analysis for the event CMVd+.

Parameter	Coefficient	Standard Error	Odds Ratio	CI	*p*
**Age at transplantation**	0.01	0.008	1.01	0.99–1.03	0.11
**s-Albumin T1**	−0.69	0.26	0.50	0.29–0.84	**0.009**
**Hb T1**	0.01	0.07	1.01	0.87–1.17	0.85
**Steroids T1**	0.0004	0.0002	1.0	1.0–1.0	0.06

Note: CI: confidence interval; s-: serum; Hb: hemoglobin.

**Table 7:** Multivariate analysis for the event CMVd+ including CMV serology at the time of transplantation.

Parameter	Coefficient	Standard Error	Odds Ratio	CI	*p*
**Age at transplantation**	0.014	0.009	1.014	0.99–1.03	0.122
**s-Albumin T1**	−0.718	0.271	0.487	0.28–0.82	**0.008**
**Hb T1**	−0.0005	0.075	0.999	0.86–1.15	0.994
**Steroids T1**	0.0003	0.0002	1.0004	0.999–1.00	0.084
**CMV serology D+/R-**	0.77	0.30	2.16	1.18–3.95	**0.01**

Note: CI: confidence interval; s-: serum; Hb: hemoglobin; CMV: cytomegalovirus; D: donor; R: recipient.

**Table 8:** Evaluation of graft failure and combined outcomes was found in relation to CMVd status.

Parameters	CMVd+ (N = 225)	CMVd− (N = 280)	*p*
**D (%)**			0.20
No	204	243	
Yes	21	37	
**sCr > 50% + D+**			0.4672
No	183	220	
Yes	42	60	
**eGFRr > 50% + D+**			0.0751
No	197	228	
Yes	28	52	
**eGFR: Variation/year of FU** (mL/min/year)	−0.7 (−1.2/+1.1)	−0.9 (−1.6/+1.1)	0.66

Note: N: number; eGFRr: estimated glomerular filtration rate reduction; sCr: serum creatinine; D: dialysis; D+: dialysis restart.

## Data Availability

The data presented in this study are available on request from the corresponding author. The data are not publicly available due to privacy issues.
